# Long-term outcomes of IMiD-based trials in patients with immunoglobulin light-chain amyloidosis: a pooled analysis

**DOI:** 10.1038/s41408-019-0266-9

**Published:** 2020-01-08

**Authors:** Rahma Warsame, Betsy LaPlant, Shaji K. Kumar, Kristina Laumann, Gabriela Perez Burbano, Francis K. Buadi, Morie A. Gertz, Robert A. Kyle, Martha Q. Lacy, David Dingli, Nelson Leung, Suzanne R. Hayman, Prashant Kapoor, Yi L. Hwa, Amie Fonder, Miriam Hobbs, Wilson I. Gonsalves, Taxiarchis Kourelis, John Lust, Stephen J. Russell, Steven Zeldenrust, Yi Lin, Eli Muchtar, Ronald S. Go, S. Vincent Rajkumar, Angela Dispenzieri

**Affiliations:** 1 Division of Hematology, Mayo Clinic, Rochester, MN USA; 2 Division of Biostatistics and Informatics, Mayo Clinic, Rochester, MN USA; 3 Division of Nephrology, Mayo Clinic, Rochester, MN USA

**Keywords:** Randomized controlled trials, Haematological cancer

## Abstract

Rarity of light-chain amyloidosis (AL) makes randomized studies challenging. We pooled three phase II studies of immunomodulatory drugs (IMiDs) to update survival, toxicity, and assess new response/progression criteria. Studies included were lenalidomide-dexamethasone (Len-Dex) (*n* = 37; years: 2004–2006), cyclophosphamide-Len-Dex (*n* = 35; years: 2007–2008), and pomalidomide-Dex (*n* = 29; years: 2008–2010) trial. Primary endpoint was hematologic response. Overall survival (OS) was calculated from registration to death and progression-free survival (PFS) was calculated from registration to progression or death. Hematologic, cardiac, and renal response/progression was assessed using the modern criteria. Analysis included 101 patients, with a median age of 65 years, 61% male, 37 newly diagnosed (ND), and 64 relapsed/refractory (RR). Median follow-up was 101 months (range 17–150) and 78% of patients died. OS and PFS for pooled cohort were 31 and 15 months, respectively. Forty-eight patients achieved a hematologic response; for ND, 10 patients (28%) achieved ≥VGPR (very good partial response) and 8 (14%) among the RR. Only cardiac stage was prognostic for OS. Common grade ≥3 toxicities were hematologic, fatigue, and rash, and were similar among studies. Hematologic and renal responses occurred more frequently and rapidly using modern response criteria; cardiac response was less frequent but occurred quickly. IMiDs can result in long progression-free intervals/survival with tolerable toxicities. The new response/progression criteria were rapid and allows for tailoring therapy.

## Introduction

Light-chain amyloidosis (AL) is a rare plasma cell disorder characterized by insoluble protein deposition on tissue-causing multi-organ dysfunction and death. Incidence of AL is estimated to be 1.2 per 100,000 person years^1^. Although rare over the past decade, there have been dramatic improvements in survival of AL patients^2^. Much of this is attributed to improvement in diagnosis, treatment, and updating the clinical response and progression criteria for hematologic, cardiac, and renal involvement^3,4^. Treatment is aimed at eradication of the underlying clonal plasma cell to stop production of amyloid fibrils, which is necessary for potential organ response and improvement.

The rarity of this disorder can make clinical trials challenging, and when conducted usually have relatively small sample sizes. Many beneficial treatments used to date have been borrowed from successful multiple myeloma regimens. These include alkylators, proteosome inhibitors, high-dose chemotherapy followed by autologous stem cell transplantation, and immunomodulatory drugs (IMiDs). The IMiDs such as thalidomide, lenalidomide, and pomalidomide are active agents in AL and have been studied in various phase I and II studies with generally small sample sizes (*n* = 13–84) and response rates ranging from 41 to 68%^5–10^. These agents are used frequently in treating AL patients, but follow-up for the long-term outcomes of trials was relatively short. Three phase II studies were performed at Mayo Clinic (Rochester, Scottsdale, Jacksonville) to test the efficacy, toxicity, and response of single agent and/or combinations of IMiDs. These studies were pooled to update the long-term outcomes and to gain an understanding on the durability and effectiveness of this class of therapy for AL. Lastly, we applied the new clinical response and progression criteria to our historical cohort.

## Methods

### Patients and treatment

A total of 102 AL patients enrolled in three phase II clinical studies were pooled. Written informed consent was obtained for all subjects upon the time of enrollment in the study. Only 101 were included for analysis; 1 patient withdrew consent prior to initiation of the study drug. Four patients were enrolled on more than one trial; data from the first trial enrolled was used for this analysis. The lenalidomide-dexamethasone study (Len-Dex) enrolled patients between October 2004 and July 2006 (*n* = 37); cyclophosphamide-Len-Dex study (Cy-Len-Dex) enrolled patients between December 2007 and November 2008 (*n* = 35). Both trials included patients who were newly diagnosed or had relapsed/refractory disease. The pomalidomide-Dex study (Pom-Dex) enrolled patients between November 2008 and November 2010 (*n* = 29), and this trial was for relapsed/refractory patients only. Inclusion and exclusion criteria for these studies were essentially identical, except for the following: creatinine needed to be ≤2.5 mg/dL for Pom-Dex and <3 mg/dL for Len-Dex and Cy-Len-Dex, and the Len-Dex study did not exclude based on New York Heart Association (NYHA) class. Whereas the Cy-Len-Dex and Pom-Dex studies included only patients who had NYHA class I and II. The Len-Dex study is the only one to include a performance status of 3.

The detailed methods of the treatment protocols were published previously for each of these studies^5–7,9,11,12^. Briefly, the Len-Dex study comprised patients who were treated with single-agent lenalidomide at 25 mg by mouth for 21 days followed by 7 days off therapy, following three cycles if there was no response to therapy and then dexamethasone at 40 mg was added on days 1–4 and 15–18, and treatment continued as long as there was response. The dose was amended to 15 mg during the study due to adverse events. The Cy-Len-Dex study had 4-week cycles of lenalidomide 15 mg by mouth for 21 days and 7 days off, cyclophosphamide 300 mg/mg by mouth on days 1, 8, and 15, and dexamethasone 40 mg given on days 1, 8, 15, 22, and initially continued as long as response, but when potential second malignancies were reported with lenalidomide, it was discontinued after 24 cycles. The Pom-Dex study gave patients 2 mg of pomalidomide by mouth daily for 28 days along with dexamethasone at 40 mg once weekly.

All studies were reviewed and approved by the institutional review board at Mayo Clinic, Rochester, Minnesota and conducted in accordance with the Declaration of Helsinki. All studies were registered on clinicaltrials.gov MC0484: NCT00166413; MC0685: NCT00564889; MC0789: NCT00558896. Each patient gave written informed consent to participate and all data were collected prospectively.

### Clinical end points

The primary endpoint of all the studies was confirmed hematologic response based on the 2005 International Society of Amyloidosis (ISA) criteria: complete response (CR) was defined as negative serum and urine for monoclonal protein, normal FLC ratio, and marrow <5%; partial response (PR) was defined as 50% reduction of the following: M spike if >0.5 g/dL, light chain in urine has visible peak and is >100 mg/day, or if free light chain (FLC) is >10 mg/dL^3^. Time to response was calculated as time from registration to first documentation of response. Safety and toxicity were assessed every 4 weeks using Common Terminology Criteria for Adverse Effects (CTCAE) version 3. Overall survival (OS) was calculated from time of registration of study to death; progression-free survival (PFS) was calculated from trial entry to progression or death. Updated follow-up and progression information was abstracted from patient records.

We applied the modern criteria for hematologic response based on the difference of FLC reduction (dFLC); CR is negative serum and urine immunofixation and normal FLC ratio, very good PR (VGPR) is dFLC <40 mg/L, and PR is dFLC decrease >50%. Cardiac response was defined as N-terminal pro b-type natriuretic peptide (NT proBNP) decreased by 30% and more than 300 ng/L if baseline NT proBNP was ≥650 ng/L, or improvement in NYHA class of two classes if baseline was class 3 or 4. Cardiac progression was defined as increase by 30% or >300 ng/L of NT proBNP or increase of troponin T ≥33% or ejection fraction decrease ≥10%. Renal response was ≥30% decrease in proteinuria or reduction of proteinuria below 0.5 g/24 h in the absence of renal progression. Renal progression was defined as ≥25% decrease in estimated glomerular filtration rate (eGFR)/proteinuria. New criteria for response/progression were compared to the prior ISA criteria. All progressions were confirmed with two consecutive assessments. All cycles of treatment were included for analysis; patients with cardiac or renal involvement were included in individual analyses. Hematologic analysis included only patients with baseline dFLC >50 mg/L. Renal progression to dialysis could not be analyzed because there were too few events.

### Statistical analysis

Data were frozen as of 18 July 2017. Updated patient follow-up and progression information was abstracted from patient records. Time to response, OS, and PFS were calculated using the Kaplan–Meier method. Univariate analyses were done using Cox proportional hazards model. Comparison between historical and modern staging and response/progression criteria were done with calculation of time to response, and comparing the differences. Statistical significance was defined as a *P* value <0.05.

## Results

### Patients

There were 101 patients included in total for analysis. Patient characteristics are described in Table 1. There were 39 (39%) female participants, with a median age of 65 years. The most common organ affected was the heart (70%) followed closely by kidney (61%), and more than half of patients had more than two organs involved (57%). Sixty-three percent of patients who were included in the study had relapsed disease. The most salient differences between the studies included time to diagnosis to registration, with the Cy-Len-dex being the shortest and Pom-dex being the longest (median of 1.6 versus 36 months); renal stage, with Pom-dex having nearly 80% of participants in stage I as compared to the other two studies in which ~45% of patients were renal stage I; and cardiac biomarker stage with Pom-dex having lower cardiac biomarker stage than the other two studies.

**Table 1:** Patient characteristics.

	MC0484 (*N* = 37)	MC0685 (*N* = 35)	MC0789 (*N* = 29)	Total (*N* = 101)
Age				
Median	64.0	64.0	66.0	65.0
Range	44.0–88.0	44.0–82.0	52.0–82.0	44.0–88.0
Gender				
Male	26 (70.3%)	19 (54.3%)	17 (58.6%)	62 (61.4%)
Vital status				
Alive	3 (8.1%)	13 (37.1%)	6 (20.7%)	22 (21.8%)
Dead	34 (91.9%)	22 (62.9%)	23 (79.3%)	79 (78.2%)
Months of follow-up				
Median	144.4	102.1	87.7	101.2
Range	139.2–149.7	16.7–113.3	71.7–101.6	16.7–149.7
Progression				
No progression	21 (56.8%)	13 (37.1%)	16 (55.2%)	50 (49.5%)
Progression	16 (43.2%)	22 (62.9%)	13 (44.8%)	51 (50.5%)
Previous treatment				
Yes	24 (64.9%)	11 (31.4%)	29 (100.0%)	64 (63.4%)
No	13 (35.1%)	24 (68.6%)	0 (0.0%)	37 (36.6%)
Number of prior planned regimens				
Median	1.5	1.0	2.0	1.5
Range	1.0–4.0	1.0–2.0	1.0–8.0	1.0–8.0
Prior transplant	13 (35.1%)	7 (20.0%)	14 (48.3%)	34 (33.7%)
Autologous	13	7	14	34
Allogeneic	0	0	0	0
Disease and baseline labs				
Months from diagnosis to on study	*N* = 37	*N* = 35	*N* = 28	*N* = 100
Median	8.9	1.6	36.2	7.9
Range	0.4–237.3	0.1–128.7	0.9–104.1	0.1–237.3
Dominant disease				
Heart	14 (37.8%)	10 (28.6%)	18 (62.1%)	42 (41.6%)
Peripheral nerve neuropathy	1 (2.7%)	1 (2.9%)	2 (6.9%)	4 (4.0%)
Autonomic nerve neuropathy	0 (0.0%)	0 (0.0%)	1 (3.4%)	1 (1.0%)
Skin	0 (0.0%)	3 (8.6%)	0 (0.0%)	3 (3.0%)
Kidney	14 (37.8%)	17 (48.6%)	3 (10.3%)	34 (33.7%)
Macroglossia	1 (2.7%)	0 (0.0%)	0 (0.0%)	1 (1.0%)
Stomach	1 (2.7%)	0 (0.0%)	0 (0.0%)	1 (1.0%)
Liver	1 (2.7%)	4 (11.4%)	0 (0.0%)	5 (5.0%)
Soft tissue	4 (10.8%)	0 (0.0%)	0 (0.0%)	4 (4.0%)
Gastrointestinal/small bowel	0 (0.0%)	0 (0.0%)	1 (3.4%)	1 (1.0%)
Other	1 (2.7%)	0 (0.0%)	1 (3.4%)	2 (2.0%)
Skin/soft tissue	0 (0.0%)	0 (0.0%)	3 (10.3%)	3 (3.0%)
Number of organs involved (heart, kidney, liver, or nerve)				
0	3 (8.1%)	1 (2.9%)	3 (10.3%)	7 (6.9%)
1	13 (35.1%)	12 (34.3%)	12 (41.4%)	37 (36.6%)
2	15 (40.1%)	18 (51.4%)	11 (37.9%)	44 (43.6%)
3	6 (16.2%)	3 (8.6%)	3 (10.3%)	12 (11.9%)
4	0 (0.0%)	1 (2.9%)	0 (0.0%)	1 (1.0%)
Median	2.0	2.0	1.0	2.0
Range	0.0–3.0	0.0–4.0	0.0–3.0	0.0–4.0
Proteinuria (g/24 h)	*N* = 36	*N* = 35	*N* = 28	*N* = 99
Median	1.78	2.0	0.2	1.0
Range	0.03–14.3	0.03–14.0	0.0–9.4	0.02–14.3
Difference between involved and uninvolved FLC (mg/dL)	*N* = 37	*N* = 35	*N* = 28	*N* = 100
Median	22.6	23.3	15.5	21.6
Range	2.3–276.8	0.75–180.8	3.2–705.8	0.75–705.8
Creatinine value (mg/dL)				
Median	1.3	1.2	1.0	1.2
Range	0.7–2.9	0.5–2.8	0.7–2.4	0.5–2.9
eGRF^a^ (mL/min per 1.73 m^2^)				
Median	53.9	65.2	66.6	64.8
Range	22.8–108.8	18.7–126.7	23.1–122.3	18.7–126.7
Renal staging (proteinuria and eGFR)	*N* = 36	*N* = 35	*N* = 28	*N* = 99
Stage I (no risk factor	17 (47.2%)	16 (45.7%)	22 (79.6%)	55 (55.6%)
Stage II (1 risk factor)	11 (30.6%)	14 (40.0%)	5 (17.9%)	30 (30.3%)
Stage III (2 risk factors)	8 (22.2%)	5 (14.3%)	1 (3.6%)	14 (14.1%)
Serum troponin (ng/mL)				
Median	0.03	0.02	0.01	0.02
Range	0.01–0.55	0.01–0.22	0.01–0.12	0.01–0.55
NT proBNP (μg)	*N* = 37	*N* = 35	*N* = 28	*N* = 100
Median	2020.0	1349.0	1856.0	1856.0
Range	103.0–42844.0	0.0–25926.0	120.0–36498.0	0.0–42844.0
IVS (mm)				
Median	13.0	14.0	15.0	14.0
Range	(9.0–24.0)	(9.0–23.0)	(9.0–22.0)	(9.0–24.0)
LVEF (%)				
Median	61.0	62.0	60.0	61.0
Range	22.0–72.0	35.0–75.0	37.0–80.0	22.0–80.0
NY heart class	*N* = 37	*N* = 35	*N* = 25	*N* = 97
I	18 (48.6%)	17 (48.6%)	15 (60.0%)	50 (51.5%)
II	14 (37.8%)	18 (51.4%)	10 (40.0%)	42 (43.3%)
III	5 (13.5%)	0 (0.0%)	0 (0.0%)	5 (5.2%)
Mayo cardiac staging (2004)	*N* = 37	*N* = 35	*N* = 28	*N* = 100
Stage I	6 (16.2%)	8 (22.9%)	1 (3.6%)	15 (15.0%)
Stage II	16 (43.2%)	12 (34.3%)	20 (71.4%)	48 (48.0%)
Stage IIIa	9 (24.3%)	7 (20.0%)	4 (14.3%)	20 (20.0%)
Stage IIIb	6 (16.2%)	8 (22.9%)	3 (10.7%)	17 (17.0%)
Mayo staging (2012)				
Stage I	6 (16.2%)	5 (14.3%)	7 (25.9%)	18 (18.2%)
Stage II	11 (29.7)	14 (14%)	11 (40.7%)	36 (36.4%)
Stage III	10 (27%)	5 (14.3%)	5 (18.5%)	20 (20.2%)
Stage IV	10 (27%)	11 (31.4%)	4 (14.8%)	25 (25.3%)
Missing	0	0	2	2

^a^eGFR is estimated GFR calculated by the abbreviated MDRD equation: 186 × (creatinine/88.4) − 1.154 × (age) − 0.203 × (0.742 if female) × (1.210 if black).

### Survival and prognosis

**Fig. 1:**
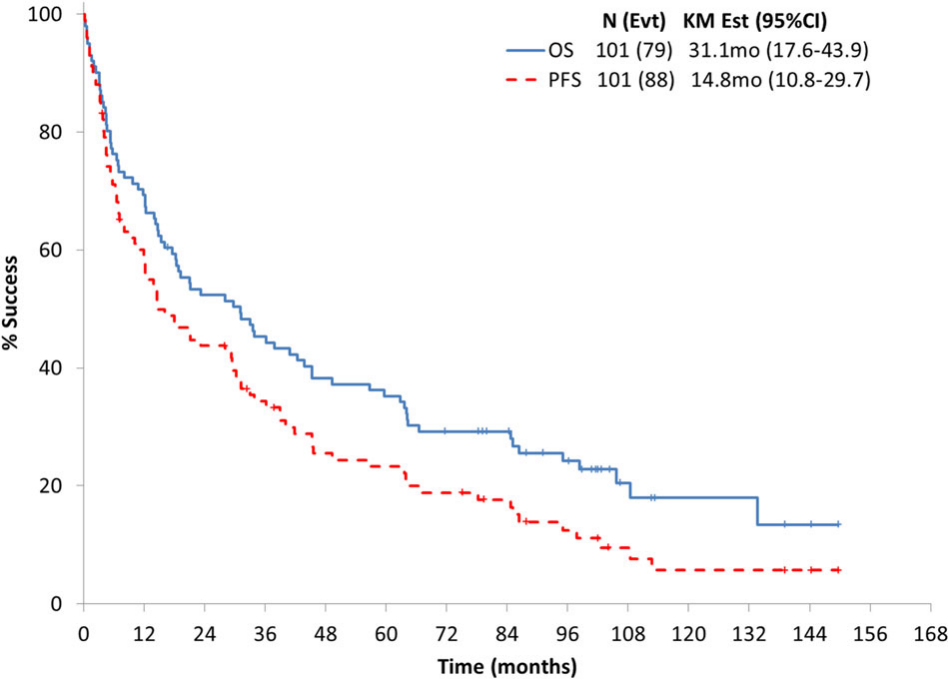
Overall survival and progression-free survival for pooled cohort.

**Fig. 2:**
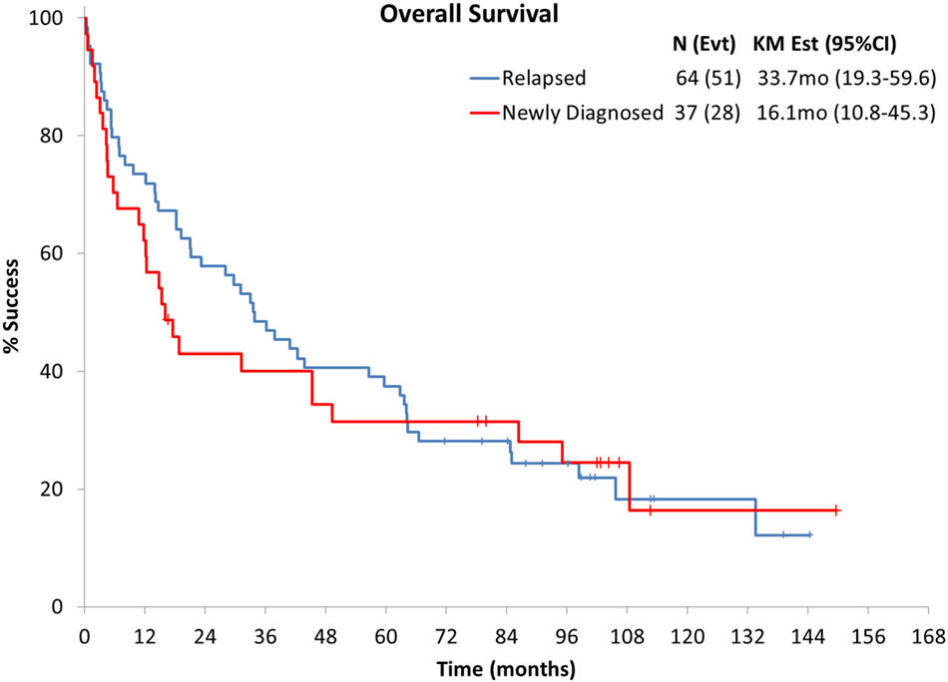
Overall survival by newly diagnosed or relapsed disease.

**Fig. 3:**
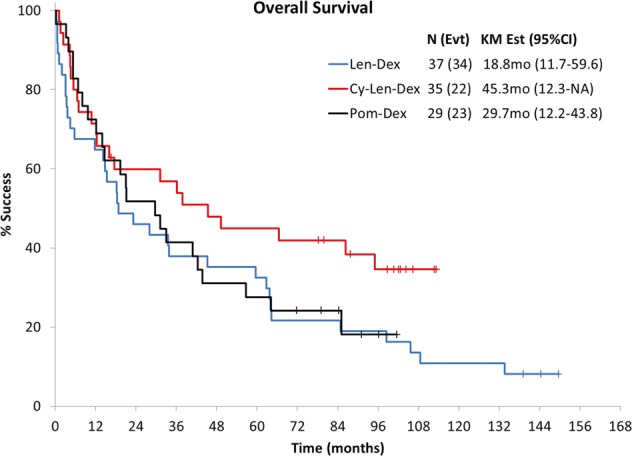
Overall survival by trial.

The median follow-up for surviving patients was 101 months (range 17–150), and 78% of patients have died. The median OS and PFS for the pooled cohort of patients were 31 months (95% confidence interval (CI): 18–44) and 15 months (95% CI: 11–30), respectively, as shown in Fig. 1. The 5-year OS rate was 35% (95% CI: 27–46) and 5-year PFS rate was 23% (95% CI: 15–34). In Fig. 2 the survival based on disease state, that is, newly diagnosed versus relapsed/refractory, is shown to be 16 versus 34 months. The long-term outcomes based on study and disease status are shown in Table 2. The updated 5-year OS rate for the Len-Dex, Cy-Len-Dex, and Pom-Dex were as follows: 32% (95% CI: 11.7–59.6), 45% (95% CI: 12.3–not reached), and 28% (95% CI: 12.2–43.8) (Fig. 3). The OS based on cardiac stage was stage I: 106 months (95% CI: 64–not reached), stage II: 35 months (95% CI: 18.3–66.5), stage IIIa: 19.6 months (95% CI: 5.5–45.2) and stage IIIb: 4 months (95% CI: 0.7–12.3); the PFS by cardiac stage was stage I: 71 months (95% CI: 39–109); stage II: 15 months (95% CI: 10–30); stage IIIa: 17.6 months (95% CI: 5.5–45); and stage IIIb: 4 months (95% CI: 0.7–6.5). This was similar with using the Mayo 2012 staging system with 85 months (95% CI: 64–not reached) for stage I, 60 months (95% CI: 36.2–105.6) for stage II, 16 months (95% CI: 12.2–49.3) for stage III, and 6 months (95% CI: 4–16) for stage IV. On univariate analysis, only cardiac stage was prognostic for OS. Prior treatment, previous IMiD, renal stage, or months from diagnosis to registration were not prognostic, so multivariate could not be performed.

**Table 2:** Hematologic, renal, and cardiac response and progression rates.

	Old criteria (per protocol)	New criteria
Hematologic		
*N*	92	92
Responders	48 (52%)	57 (62%)
CR	6	15
VGPR	12	22
PR	30	20
Renal		
*N*	62	62
Responders	15 (24%)	31 (50%)
Time to response, median (range)	161 days (28–502)	62 days (28–480)
Progression	14 (23%)	27 (44%)
Time to progression, median (range)	196 days (21–1022)	144 days (26–1230)
Cardiac		
*N*	71	71
Responders	11 (15%)	6 (8%)
Time to response, median (range)	213 days (58–995)	196 days (35–510)
Progression	10 (14%)	19 (25%)
Time to progression, median (range)	232 days (84–697)	30 days (23–396)

### Treatment response and progression criteria

Table 2 shows the response and progression rates for hematologic, renal, and cardiac involvement for historical ISA and modern criteria. There were 62 renal, 71 cardiac, and 92 hematologic patients who had complete data that could be included for analysis. In terms of hematologic response among the pooled cohort, there were more responders (62% versus 52%), higher CR (26% versus 12.5%), and VGPR rates (39% versus 25%) using the modern criteria than prior ISA guidelines. Specifically looking at the newly diagnosed (*n* = 35), 43% (15/35) versus 28% (10/35) achieved VGPR or better when new criteria are compared to historic ISA guidelines. Similarly, among the patients with relapsed and refractory disease (*n* = 57), 39% (22/57) versus 14% (8/57) achieved VGPR or better. With respect to renal response or progression, the new criteria had higher response rates (50% versus 24%) and progression rates (44% versus 23%). The time to achieve response was shorter with new criteria (62 versus 161 days) and progression (144 versus 199 days) was also shorter based on the new criteria. Among the 62 patients with renal involvement, 9 developed end-stage renal disease and required dialysis. There were fewer cardiac responses based on new criteria (8% versus 15%), although median time to obtain a response was faster (197 versus 213 days). Cardiac progression was more frequent with the new criteria than historic criteria as well (25% versus 14%). Median time to progression was faster with the new criteria (30 versus 232 days), with a median time to progression 110 days earlier. There were two patients who were responders on old criteria but were progression on new criteria, on the Pom-dex trial, and had relapsed disease. With unconfirmed progression there would have been five patients who were responders historically and considered progression on modern criteria. For patient 1, cardiac progression was confirmed at 167 days based on the new criteria, achieved a hematologic PR, but remained on the trial for an additional 272 days. Patient 2 had cardiac progression by 84 days on study, achieved a hematologic VGPR but remained on study for 644 more days until she died on study. Appendix A shows the data regarding their dates of progression, trial enrolled, survival, and time to next therapy.

### Toxicities

There were a total of seven deaths during the study (Len-Dex = 3, Cy-Len-Dex = 3, Pom-Dex = 1). The most frequent adverse events were hematologic, with neutropenia being the most common among all the studies (Len-Dex *n* = 19, Cy-Len-Dex *n* = 13, and Pom-Dex *n* = 9). Fatigue was the most frequent non-hematologic adverse event that was grade 3 or higher, with 41% for the Len-Dex, 43% for Cy-Len-Dex, and 21% for Pom-Dex studies. There were no rashes reported in Pom-Dex study, but Len-Dex and Cy-Len-Dex each had four incidents of rash. Cardiac arrhythmias occurred most frequently in the Cy-Len-Dex study *n* = 9 (24%) and least in Pom-Dex study *n* = 5 (17%). The common grade 3 or higher toxicities regardless of attribution are shown in Table 3.

**Table 3:** Listing of grade 3 + adverse events, regardless of attribution.

Adverse event^a^	Study	Grade 3	Grade 4	Grade 5
		*n* (%)	*n* (%)	*n* (%)
Hemoglobin	Len-Dex	2 (5%)	0 (0%)	0 (0%)
	Cy-Len-Dex	6 (17%)	2 (6%)	0 (0%)
	Pom-Dex	0 (0%)	1 (3%)	0 (0%)
Neutrophils/granulocytes (ANC/AGC)	Len-Dex	12 (32%)	7 (19%)	0 (0%)
	Cy-Len-Dex	6 (17%)	7 (20%)	0 (0%)
	Pom-Dex	6 (21%)	3 (10%)	0 (0%)
Platelets	Len-Dex	6 (16%)	4 (11%)	0 (0%)
	Cy-Len-Dex	6 (17%)	6 (17%)	0 (0%)
	Pom-Dex	2 (7%)	0 (0%)	0 (0%)
Hypotension	Len-Dex	2 (5%)	3 (8%)	1 (3%)
	Cy-Len-Dex	4 (11%)	1 (3%)	1 (3%)
	Pom-Dex	0 (0%)	0 (0%)	0 (0%)
Fatigue	Len-Dex	12 (32%)	3 (8%)	0 (0%)
	Cy-Len-Dex	15 (43%)	0 (0%)	0 (0%)
	Pom-Dex	5 (17%)	1 (3%)	0 (0%)
Supraventricular and nodal arrhythmia	Len-Dex	3 (8%)	2 (5%)	0 (0%)
	Cy-Len-Dex	4 (11%)	1 (3%)	0 (0%)
	Pom-Dex	3 (10%)	0 (0%)	0 (0%)
Thrombosis/thrombus/embolism	Len-Dex	1 (3%)	1 (3%)	1 (3%)
	Cy-Len-Dex	0 (0%)	4 (11%)	0 (0%)
	Pom-Dex	1 (3%)	1 (3%)	0 (0%)
Ventricular arrhythmia	Len-Dex	0 (0%)	0 (0%)	1 (3%)
	Cy-Len-Dex	1 (3%)	0 (0%)	1 (3%)
	Pom-Dex	1 (3%)	0 (0%)	0 (0%)
Rash/desquamation	Len-Dex	4 (11%)	0 (0%)	0 (0%)
	Cy-Len-Dex	4 (11%)	0 (0%)	0 (0%)
	Pom-Dex	0 (0%)	0 (0%)	0 (0%)
Dehydration	Len-Dex	1 (3%)	0 (0%)	0 (0%)
	Cy-Len-Dex	5 (14%)	0 (0%)	0 (0%)
	Pom-Dex	0 (0%)	0 (0%)	0 (0%)
Diarrhea	Len-Dex	2 (5%)	0 (0%)	0 (0%)
	Cy-Len-Dex	4 (11%)	1 (3%)	0 (0%)
	Pom-Dex	1 (3%)	0 (0%)	0 (0%)
Infection w/ normal or grade 1/2 ANC	Len-Dex	4 (11%)	0 (0%)	0 (0%)
	Cy-Len-Dex	4 (11%)	1 (3%)	0 (0%)
	Pom-Dex	2 (7%)	1 (3%)	0 (0%)
Infection with grade 3 or 4 ANC	Len-Dex	6 (16%)	1 (3%)	0 (0%)
	Cy-Len-Dex	3 (9%)	1 (3%)	1 (3%)
	Pom-Dex	3 (10%)	1 (3%)	1 (3%)
Edema: limb	Len-Dex	8 (22%)	0 (0%)	0 (0%)
	Cy-Len-Dex	8 (23%)	0 (0%)	0 (0%)
	Pom-Dex	3 (10%)	0 (0%)	0 (0%)
Potassium, serum low (hypokalemia)	Len-Dex	4 (11%)	1 (3%)	0 (0%)
	Cy-Len-Dex	2 (6%)	1 (3%)	0 (0%)
	Pom-Dex	0 (0%)	1 (3%)	0 (0%)
Syncope (fainting)	Len-Dex	7 (19%)	0 (0%)	0 (0%)
	Cy-Len-Dex	6 (17%)	0 (0%)	0 (0%)
	Pom-Dex	2 (7%)	0 (0%)	0 (0%)
Dyspnea (shortness of breath)	Len-Dex	9 (24%)	4 (11%)	0 (0%)
	Cy-Len-Dex	5 (14%)	0 (0%)	0 (0%)
	Pom-Dex	6 (21%)	0 (0%)	0 (0%)

^a^Per NCI CTCAE version 3.0.

## Discussion

AL amyloidosis is a heterogeneous disease and treatment can be a challenge because of the degree of organ involvement. Treatments have evolved over the past decade and IMiDs are increasingly used in AL patients. The ideal treatment sequence or strategy is unknown in AL and there are no prospectively randomized studies to guide clinicians on when it is best to use IMiDs. Moreover, the published phase I and phase II studies conducted have relatively limited follow-up. This study reports on a large cohort of AL patients (102 patients pooled) to showcase the efficacy of IMIDs in the treatment of AL both in newly diagnosed and relapsed disease settings with substantial median follow-up of 101 months. In the analysis of this study, patients had prolonged OS and PFS, excellent hematologic response, and toxicities that were manageable. Toxicity appeared to be lower with pomalidomide, but as a whole, this cohort had less advanced disease prior to registration than did the patients receiving lenalidomide-based regimens.

The OS for this pooled cohort was 31 months, in part due to the fact that the majority of patients were relapsed/refractory patients who had median OS of 34 months as compared to their newly diagnosed counterparts who had a median OS of 16 months. This reflects the phenomena that newly diagnosed AL patients typically have shorter survival rates than their relapsed counterparts since there is 30–40% early death rate typically observed^13^. Moreover, unlike modern-day trials, none of these trials excluded patients’ advanced stage disease (stage IIIa or IIIb). In fact, 37% of patients were stage III, among those 17% were stage IIIb. The survival observed for each of these studies is comparable to other studies with similar combinations of Len-Dex, Alkylator-Len-Dex, and Pom-Dex^12,14,15^. The three studies included in the cohort span the past 11 years, and although the Pom-Dex study is the most recent, the Cy-Len-Dex study had the best PFS (31 months). However, no conclusion can be drawn between the three studies because of the lack of a multivariate factors and heterogeneity of baseline characteristics.

This pooled study shows that hematologic response with IMiDs is high with a objective response rate of 62% using updated response criteria (52% response rate using the 2005 ISA response), and 43% of the pooled cohort of patients achieved a VGPR or CR. This is comparable to other published phase I or II studies with IMiDs with hematologic response rates ranging from 46 to 62%^8,9,12,15–17^. Considering these data include studies that began as early as 2007, including full-dose lenalidomide for some trials, a dose typically difficult for AL patients to tolerate highlights the benefit of IMiD combinations. When compared to bortezomib-containing regimens that have been reported that range from 60 to 94% in newly diagnosed, and 69 to 100% in relapsed refractory AL patients, IMiDs have a lower response rate. Thus, IMiDs are best incorporated as a treatment for relapsed disease, and bortezomib-containing agents are utilized in first-line setting if transplant ineligible, or after first relapse from transplant. Unfortunately, there are no data in amyloid literature regarding IMiD usage following proteasome inhibitor (PI) progression. However, if we extrapolate from the myeloma literature that shows that pomalidomide with or without low-dose dexamethasone had 37 and 10% objective response, respectively, after prior carfilzomib, then IMiD following PI’s is reasonable to consider in AL as well.

Renal and hematologic responses were higher when using the modern criteria as a post hoc analysis. As previously demonstrated^4,18^, the newer response criteria also detect hematologic, cardiac, and renal responses more rapidly than the older criteria. Notably, cardiac responses were less frequent using the modern criteria, and this is likely due to the increase in NT proBNP for AL patients on IMiDs and confirms the discordant response in NT proBNP than the FLC reported previously^19^. Exploring the two patients who were discordant between historic and modern cardiac response criteria, they both remained on study over a year after they would have been considered progression and continued to tolerate therapy with mild toxicity and achieve hematologic response. Although only two patients and no conclusions can be drawn, it is interesting that they gained benefit and did not experience any undue toxicity while on the trial despite meeting progression criteria.

In conclusion, IMiDs are generally well tolerated, and an effective durable therapy for patients with AL amyloidosis. It can be used in newly diagnosed, but preferably in relapsed/refractory patients who are more likely to tolerate the NT proBNP fluctuations and have already proven the pace of their disease.

## Supplementary information

Characteristics of patients who would have been considered progression on new criteria but were responders on old criteria
