# Supplementary material for: Long-term outcomes of IMiD-based trials in patients with immunoglobulin light-chain amyloidosis: a pooled analysis
Source: Blood Cancer J. 2020 Jan 8;10(1):4. doi: 10.1038/s41408-019-0266-9 (PMC6949262; doi:10.1038/s41408-019-0266-9)
Supplement: Supplementary file 1 — Characteristics of patients who would have been considered progression on new criteria but were responders on old criteria [file 41408_2019_266_MOESM1_ESM.docx]

Appendix Table: Characteristics of patients who would have been considered progression on new criteria but were responders on old criteria.

|  | Patient 1 | Patient 2 |
| --- | --- | --- |
| Age | 71 | 70 |
| Sex | Female | Female |
| Regimen | Pom-Dex | Pom-Dex |
| Disease status at enrollment | Relapsed/refractory | Relapsed/refractory |
| Baseline  NT Pro-BNP pg/mL | 3507 | 7351 |
| Time to progression or death (historical criteria), days | 441 | 635 |
| Time to progression or death (new criteria), days | 167 | 84 |
| Alive or Dead | Dead | Died on study |
| Survival since trial enrollment (days) | 578 | 635 |
| Time to next therapy since study completion (days) | 103 | Died on study |
| Major toxicity on trial | Fatigue, mood swings attributed to dexamethasone  Grade 3 syncope possibly attributed to IMiD | Fatigue, edema, proximal myopathy most attributed to dexamethasone |
| Old Hematologic Response | PR | VGPR |
| New hematologic response | VGPR | CR |
